# A retrospective cohort study using a national surveillance questionnaire to investigate the characteristics of maternal venous thromboembolism in Japan in 2018

**DOI:** 10.1186/s12884-021-03993-1

**Published:** 2021-07-17

**Authors:** Mamoru Morikawa, Tomoko Adachi, Atsuo Itakura, Masafumi Nii, Yasushi Nakabayashi, Takao Kobayashi

**Affiliations:** 1 Department of Obstetrics and Gynecology, Kansai Medical University, Shinmachi 2-5-1, Hirakata City, Osaka, 573-1010 Japan; 2 Department of Obstetrics and Gynecology, Aiiku Hospital, Tokyo, Japan; 3 Department of Obstetrics and Gynecology, Juntendo University Graduate School of Medicine, Tokyo, Japan; 4 Department of Obstetrics and Gynecology, Mie University Graduate School of Medicine, Tsu, Japan; 5 Department of Obstetrics and Gynecology, Nakabayashi Hospital, Tokyo, Japan; 6 Department of Obstetrics and Gynecology, Hamamatsu Medical Center, Hamamatsu, Japan

**Keywords:** Venous thromboembolism, Pulmonary thromboembolism, Antepartum, Postpartum, Pregnancy

## Abstract

**Background:**

In Japan, the numbers of deliveries by women of older maternal age and women with overweight or obesity have recently increased. Since 2008, the guidelines and practices to prevent the maternal venous thromboembolism (VTE) have been recommended antepartum and postpartum thromboprophylaxis for each risk level of VTE. This study aimed to clarify the incidence and characteristics (type of VTE and thromboprophylaxis) of VTE in pregnant women in Japan to reduce the rate of mortality from VTE

**Methods:**

Of 2299 institutions sent the surveillance questionnaire, 666 (29.0%) responded, and data from 295,961 women who gave birth in those institutions in 2018 were analyzed. We calculated the incidence and characteristics of VTE before and after the deliveries.

**Results:**

At the responding institutions, 243 women (0.082%) had VTE in 2018. In 2018, deep vein thrombosis was significantly more common (0.0053%) than pulmonary thromboembolism (0.0019%; *p* < 0.0001). The incidence of antepartum VTE (0.0055%) was significantly higher than that of postpartum VTE (0.0026%; *p* < 0.0001). The incidence of VTE after cesarean Sect. (0.0074%) was significantly higher than that after vaginal delivery (0.0012%; *p* < 0.0001). Of the women with VTE, 4 (1.6%) died.

**Conclusions:**

Among the women thought to have a low risk of VTE during the antepartum period, and especially women who had a vaginal delivery, the actual incidence of VTE might have increased in Japan.

**Supplementary Information:**

The online version contains supplementary material available at 10.1186/s12884-021-03993-1.

## Background

Pregnant women and women who have recently given birth are in states of hypercoagulability [1] and are thus at increased risk of venous thromboembolism (VTE) [2], one of the leading causes of maternal death in developed countries [3, 4].

Women who have delivered by cesarean section are at increased risk of VTE [5–7]. In the United States, the incidence of VTE among pregnant and postpartum women is four to five times higher than that among nonpregnant women [8], the incidence of VTE was 1.72 per 1000 deliveries, with 1.1 deaths per 100,000 from 2000 to 2001 [9], and the deaths from pulmonary embolism (PE) accounted for 9.2% of all pregnancy-related deaths, or approximately 1.5 deaths per 100,000 live births, from 2004 to 2014 [10].

In Japan [3], the mortality induced by VTE was 7.1% (24) of all 338 maternal deaths from 2010 to 2016, according to the maternal death registration system established by the Japan Association of Obstetricians and Gynecologists (JAOG) and the Maternal Death Exploratory Committee [3]. The incidence of maternal deaths from VTE was 0.33 deaths per 100,000 live births (7,175,733 women) from 2010 to 2016 in Japan.

In Japan, deliveries by women of older maternal age and by women with overweight or obesity have recently increased; thus, the risk of VTE may have increased. On the other hand, since 2008, the Japan Society of Obstetrics and Gynecology (JSOG) and JAOG have recommended guidelines and practices to prevent the maternal VTE [11, 12], and so the incidence of VTE onset in Japan might have decreased since then.

The goal of this study was to clarify the incidence and characteristics of VTE (type of VTE and thromboprophylaxis), and the relationship between them in 2018 in Japan.

## Methods

### Study design

We conducted a cohort study with a surveillance questionnaire. In May 2019, questionnaires were sent to all 2299 hospitals or maternity clinics listed as institutions with obstetric facilities by the JSOG on December 31, 2018. The institutions’ chairpersons, chiefs, or their substitutes responded to the questionnaire by mail.

The surveillance questionnaire was designed by the JSOGNH and had four question categories: (1) the types and specialties of the institutions, (2) the methods used to prevent maternal VTE onset, (3) the incidence of maternal VTE (e.g., case numbers, types of VTE, period of VTE onset), and (4) the outcomes (mortality induced by VTE) among pregnant women with VTE onset in 2018. The response from hospitals or maternal clinics that had no deliveries in 2018 was excluded.

The recommendations for thromboprophylaxis in Japan [11] are shown in Table 1. These were determined according to the modified guidelines of the Royal College of Obstetricians and Gynecologists [13] and of the American College of Chest Physicians [14, 15].

**Table 1:** Recommendations for thromboprophylaxis in Japan

Antepartum thromboprophylaxis					
	Group	Risk of incidence	State during pregnancy	Thromboprophylaxis (heparin administration)	Level of recommendation
1)	1	High	Standard	Perform	B
2)	2	Moderate	Standard	Consider	B
3)	2	Moderate	Operation	Perform	B
4)	3	Low	Standard	Consider	C
**Postpartum thromboprophylaxis in Japan**					
	Group	Risk of incidence	I. Thromboprophylaxis (heparin administration) II. Intermittent pneumatic compressions		Level of recommendation
1)	1	High	Perform I or I + II		B
2)	2	Moderate	Perform I or II		B
3)	3	Low	Consider I or II		C

Level A and B treatment are regarded as current standard care practices in Japan. Level A recommendations are stronger than level B recommendations. Informed consent is therefore required when maternity service providers do not provide care corresponding to level A or B recommendations

Level C treatment consists of possible options that may favorably affect the outcome but for which it is unclear whether the possible benefits outweigh the possible risks. Thus, care corresponding to level C recommendations is not necessarily provided

All thromboembolisms were classified as one of four types: (1) DVT, (2) pulmonary thromboembolism (PE), (3) other vein thrombosis (other VT), and (4) arterial thromboembolism (ATE). The combination of PE with DVT was classified as PE. Other VTs were defined as thrombosis of more superficial veins, such as thromboembolisms of veins in the arm, ovary, colon, and brain.

In the present study, “VTE” was defined as “DVT or/and PE,” and the simultaneous occurrence of “DVT and PE” was defined as “PE.”

Time of onset was classified as antepartum or postpartum (the latter of which included the period during labor). Operations during pregnancy were defined as invasive procedures in the hospital, e.g., cervical cerclage, laparotomy, or laparoscopic ovarian cystectomy. Delivery mode was classified as vaginal delivery or cesarean section.

In the pregnant women who died, autopsy imaging (or autopsy) was performed to diagnose the cause of death.

### Statistical analyses

Data were calculated as frequencies. JMP Pro, version 14.0 (SAS Institute Inc., Cary, NC, USA), was used to perform the statistical analyses. Response: We had edited to Statistical analyses as follow: The Tukey–Kramer test was used to compare multiple categorical variables. Fisher’s exact test was used to compare each of the categorical variables after the significant difference by Tukey–Kramer was noted. In all analyses, a *p*-value of less than 0.05 indicated statistical significance.

### Ethics approval and consent to participate

This study was approved by the Institutional Review Board of Hokkaido University Hospital (018–280), Hokkaido, Japan. It was performed in compliance with the Declaration of Helsinki. The requirement for informed consent was exempted by the Review Board of Hokkaido University Hospital that approved this study.

### Patient and public involvement

None of the patients were involved in devising the research questions, outcome measures, or plans for recruitment, and in the design or implementation of the study. None of the patients were asked to advise on the interpretation or writing of the results. We have no plans to disseminate the research study results to the study participants or the relevant patient community.

### Availability of data

The datasets generated and analyzed during the present study are not publicly available because taking them out to other facilities is restricted by the institutional review board but are available from the corresponding author on reasonable request.

## Results

### Participants

**Fig. 1:**
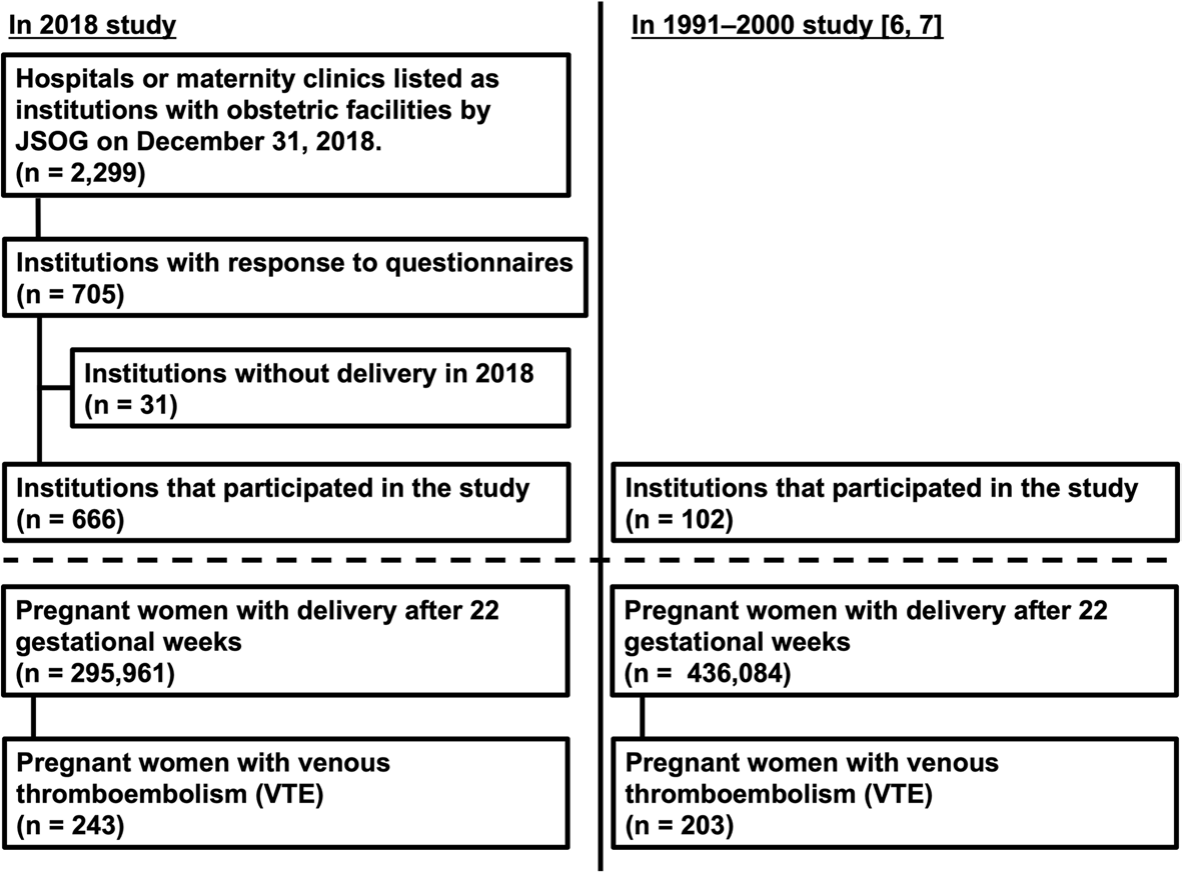
Flowchart of the 1991–2000 and 2018 studies

The flowchart of the 2018 study is shown in Fig. 1. Of the 2299 institutions that received the surveillance questionnaire, 705 (30.7%) responded to the questionnaires, of which 39 (5.5%) had no deliveries in 2018 and were thus excluded from the study. The final number of institutions that participated in this study was 666 (29.0%). At these institutions, 295,961 women delivered after 22 gestational weeks; their infants represented 32.2% of all Japanese births in 2018.

### Characteristics of thromboprophylaxis

Of the 295,961 women, 67,752 (22.9%) delivered by cesarean section. Routine thromboprophylaxis, both antepartum and postpartum, is described in Table 2. In accordance with the recommendation of thromboprophylaxis in Japan for pregnant women at high risk for VTE [11, 12], unfractionated heparin (UFH) or low-molecular-weight heparin (LMWH) was routinely administered by 316 (47.4%) institutions before the women gave birth and by 382 (57.4%) institutions after; compression stockings were routinely applied by 326 (48.9%) institutions before the women gave birth and by 396 (59.5%) institutions after; and intermittent pneumatic compressions were applied by 162 (24.3%) institutions before the women gave birth and by 353 (53.0%) institutions after. The number of institutions in which UFH or LMWH was routinely administered and in which intermittent pneumatic compressions were applied to prevent postpartum VTE was significantly higher than those applying these measures to prevent antepartum VTE (*p* < 0.0001).

**Table 2:** Characteristics of the 666 institutions that participated in the study

Types of institutions	
Perinatal medical centers	184 (27.6%)
General hospitals with obstetrics	170 (25.5%)
Maternal clinics with beds	312 (46.8%)
**Available staff/services**	
Anesthesiologists^a^	304 (45.6%)
Pediatricians^a^	336 (50.5%)
MRI, CT, or both	323 (48.5%)
**Routine antepartum thromboprophylaxis for women at high risk for thromboembolism**	391 (58.7%)
Administration of UFH or LMWH	316 (47.4%)
Application of compression stockings	326 (48.9%)
Application of intermittent pneumatic compressions	162 (24.3%)
**Routine antepartum thromboprophylaxis after surgery**	456 (68.5%)
Administration of UFH or LMWH	290 (43.5%)
Application of compression stockings	416 (62.5%)
Application of intermittent pneumatic compressions	332 (49.8%)
**Routine postpartum thromboprophylaxis for women at high risk for thromboembolism**	444 (66.7%)
Administration of UFH or LMWH	382 (57.4%)
Application of compression stockings	396 (59.5%)
Application of intermittent pneumatic compressions	353 (53.0%)
**Outcomes of all deliveries**	
Overall	295,961
Cesarean sections	67,752 (22.9%)
Maternal deaths	20 (0.0068%)

*MRI* Magnetic resonance imaging, *CT* Computed tomography, *VTE* Venous thromboembolism, *UFH* Unfractionated heparin, *LMWH* Low-molecular-weight heparin, ^a^ Working exclusively at each institution

### Characteristics of the pregnant women with venous thromboembolism

In this study, of the 295,961 women who delivered after 22 gestational weeks, 243 (0.082%) had VTE. Of the 243 women with VTE, 157 (64.6%) had DVTs, 56 (23.0%) had PEs, 26 (10.7%) had other VTs, and 4 (1.6%) had ATEs (Table 3). Among 295,961 women, the incidences of DVT, PE, other VT, and ATE were 53.0, 18.9, 8.8, and 1.4 per 100,000 women, respectively. The incidence of DVT was significantly higher than those of PE, other VT, and ATE (all *p* < 0.0001); the incidence of PE was significantly higher than those of other VT (*p* = 0.0009) and ATE (*p* < 0.0001); and the incidence of other VT was significantly higher than that of ATE (*p* < 0.0001).

**Table 3:** Relationship between the types and time of thromboembolism onset/maternal deaths from thromboembolism

	**Overall** **(n = 243, 100%)**	**A. DVT** **(n = 157, 64.6%)**	B. PE* (n = 56, 23.0%)	**C. Other VT** **(n = 26, 10.7%)**	**D. ATE** **(n = 4, 1.6%)**	***p*-value** ** < *0.05***
***Thromboembolism onset***						
**Overall**	243 (100%)	157 (100%)	56 (100%)	26 (100%)	4 (100%)	*A vs. B: 0.0044*
Antepartum	165 (67.9%)	125 (79.6%)	20 (35.7%)	16 (61.5%)	4 (100%)	*A vs. B:* < *0.0001* *B vs. C:* *0.0339*
Antepartum with operation	18 (7.4%)	11 (7.0%)	6 (10.7%)	0 (0.0%)	1 (20.0%)	*NS*
Postpartum	79 (32.5%)	32 (20.4%)	36 (64.3%)	10 (38.5%)	0 (0.0%)	*A vs. B:* < *0.0001* *B vs. C:* *0.0339*
Cesarean section	50 (20.6%)	17 (10.8%)	26 (46.4%)	7 (26.9%)	0 (0.0%)	*A vs. B:* *0.0175*
*Maternal deaths from thromboembolism*						
**Overall**	4 (1.6%)	0 (0.0%)	4 (7.1%)	0 (0.0%)	0 (0.0%)	*A vs. B:* *0.0044*
Antepartum	1 (0.4%)	0 (0.0%)	1 (1.8%)	0 (0.0%)	0 (0.0%)	NS
Antepartum with operation	0 (0.0%)	0 (0.0%)	0 (0.0%)	0 (0.0%)	0 (0.0%)	NS
Postpartum	3 (1.2%)	0 (0.0%)	3 (1.2%)	0 (0.0%)	0 (0.0%)	*A vs. B:* *0.0175*
Cesarean section	3 (1.2%)	0 (0.0%)	3 (1.2%)	0 (0.0%)	0 (0.0%)	*A vs. B:* *0.0175*

*DVT* Deep vein thrombosis, *PE* Pulmonary thromboembolism, *Other VT* Other vein thrombosis, *ATE* Arterial thromboembolism, *NS* Not significant

^*^Venous thromboembolism was DVT and/or PE. The simultaneous occurrence of DVT and PE was defined as PE

Of the 243 women with maternal VTE, 165 (67.9%) had antepartum VTE and 78 (32.1%) had postpartum VTE. Among 295,961 women, the incidence of antepartum VTE was significantly higher than that of postpartum VTE (Table 3). Of 165 women with maternal antepartum VTE, 18 (10.9%) had perioperative antepartum VTE. Among 78 women with postpartum VTE, the disorder developed in 50 (64.1%) after cesarean section and in 28 (35.9%) after vaginal delivery. The incidence of VTE was significantly higher after cesarean section than after vaginal delivery (Table 3).

### Relationships between the types and onset of thromboembolism

The characteristics of the period of VTE onset are summarized in Table 3. The odds ratio (95% confidence interval [CI]) of VTE in the 2018 study are summarized in Table 4.

**Table 4:** Odds ratio (95% CI) of venous thromboembolism

VTE-1*	Incidence rate-1	VTE-2*	Incidence rate-2	Odds ratio (95% CI)	*p-value*
**In 2018 study**					
Antepartum DVT	125/157 (79.6%)	Postpartum DVT	32/157 (20.4%)	3.91 (2.65–1.72)	< *0.0001*
Postpartum PE	36/56 (64.3%)	Antepartum PE	20/56 (35.7%)	1.18 (1.04–3.11)	*0.0440*
***Antepartum***					
Perioperative PE or VTE during pregnancy	6/18 (33.3%)	Other PE or VTE during pregnancy	14/133 (9.5%)	4.75 (1.54–14.6)	*0.0108*
Perioperative PE during pregnancy	6/20 (30.0%)	Perioperative DVT during pregnancy	11/125 (8.8%)	4.44 (1.42–13.9)	*0.0150*
***Postpartum***					
PE after cesarean section	26/67752 (0.038%)	DVT after cesarean section	17/67752 (0.025%)	1.53 (0.83–2.82)	*0.2220*
DVT after cesarean section	17/67752 (0.025%)	DVT after vaginal delivery	15/229209 (0.0065%)	3.82 (1.91–7.65)	*0.0002*
PE after cesarean section	26/67752 (0.038%)	PE after vaginal delivery	10/229209 (0.0044%)	7.86 (2.03–30.4)	*0.0020*
**In 1991–2000 studies**					
Antepartum DVT	64/127 (50.4%)	Postpartum DVT	63/127 (49.6%)	1.03 (0.63–1.69)	*1.000*
Postpartum PE	59/76 (77.6%)	Antepartum PE	17/76 (22.4%)	3.47 (2.02–5.95)	< *0.0001*
***Postpartum***					
DVT after cesarean section	35/87382 (0.040%)	DVT after vaginal delivery	28/348702 (0.008%)	4.99 (3.04–8.20)	< *0.0001*
PE after cesarean section	50/87382 (0.057%)	PE after vaginal delivery	9/348702 (0.003%)	22.2 (10.9–45.1)	< *0.0001*

*CI* Confidence interval, *VTE* Venous thromboembolism, *DVT* Deep vein thrombosis, *PE* Pulmonary thromboembolism

^*^VTE was DVT and/or PE. The simultaneous occurrence of DVT and PE was defined as PE

The postpartum incidence of PE (36/56 [64.3%]) was significantly higher than those of DVT (32/157 [20.4%]; *p* < 0.0001) and other VT (10/26 [38.5%]; *p* = 0.0339). The postpartum incidence of DVT was similar to that of other VT (*p* = 0.0745). In all four women with ATE, the onset was before delivery. Among the 165 women with antepartum VTE, the incidence of perioperative PE during pregnancy (6/20 [30.0%]) was higher than that of perioperative DVT (11/125 [8.8%]; p = 0.0150). In particular, after cesarean section, the incidence of PE (26/56 [46.4%]) was significantly higher than that of DVT (17/157 [10.8%]; *p* < 0.0001).

### Relationship between the administration of UFH or LMWH and the incidence of venous thromboembolism

Table 5 shows the relationship between the administration of unfractionated heparin (UFH) or LMWH and incidence of venous thromboembolism. The frequencies of the administration of UFH or LMWH among the women who had VTE and PE at/after vaginal delivery were significantly lower than those among women who had VTE and PE at/after cesarean section (p = 0.0002 and p = 0.0013), respectively.

**Table 5:** The relation between administration of UFH or LMWH and incidence of VTE in the 2018 study

		VTE*		DVT		PE	
		Overall	With administration of UFH or LMWH	Overall	With administration of UFH or LMWH	Overall	With administration of UFH or LMWH
Ante-partum	Overall	145 (100%)	90 (62.1%)	125 (100%)	79 (63.2%)	20 (100%)	11 (55.0%)
	With operation	17 (100%)	8 (47.1%)	11 (100%)	6 (54.5%)	6 (100%)	2 (33.3%)
	Without operation	128 (100%)	82 (64.1%)	114 (100%)	73 (64.0%)	14 (100%)	9 (64.3%)
	*p-value*		*0.1921*		*0.5313*		*0.3359*
Post-partum	Overall	68 (100%)	51 (75.0%)	32 (100%)	19 (59.4%)	36 (100%)	32 (88.9%)
	Cesarean section	43 (100%)	34 (79.1%)	17 (100%)	13 (76.5%)	26 (100%)	21 (80.8%)
	Vaginal delivery	25 (100%)	8 (32.0%)	15 (100%)	6 (40.0%)	10 (100%)	2 (20.0%)
	*p-value*		*0.0002*		*0.0702*		*0.0013*

*VTE* Venous thromboembolism, *DVT* Deep vein thrombosis, *PE* Pulmonary thromboembolism, *UFH* Unfractionated heparin, *LMWH* Low-molecular-weight heparin

^*^, *VTE was DVT and/or PE. The simultaneous occurrence of DVT and PE was defined as PE

### Characteristics of death from venous thromboembolism

The characteristics of the deaths from VTE are listed in Tables 2 and 3. In this study, of the 295,961 women who delivered after 22 gestational weeks, 20 (0.0068%) died, of whom 18 (90.0%) died in perinatal medical centers (data was not shown).

Of 243 women with thromboembolism, 4 (1.6%) died. The mortality rate among women with maternal thromboembolism was significantly higher than that among women without it (16/295,718 [0.0054%]; *p* < 0.0001). All four women who died of VTE had PE. Of the 165 women with antepartum VTE, 1 (0.61%) died, and of the 78 women with postpartum VTE, 3 (3.8%) died; in terms of the 243 with thromboembolism, these frequencies were similar (*p* = 0.0986). Of the 18 women (7.4%) with perioperative antepartum VTE, none died. Of the 50 women (20.6%) who had VTE after cesarean section, 3 (6.0%) died. Of the 4 women who died, 3 died at perinatal medical centers and 1 died at a maternal clinic with beds (data not shown).

## Discussion

Our findings emphasized the following three points: (1) the incidence rates of postpartum PE and DVT onsets were significantly higher and lower than those during pregnancy, respectively; (2) the incidence rate of PE in association with cesarean section was significantly higher than that of DVT; (3) of the 243 women with VTE, 4 (1.6%) died, all of whom had PE.

To the best of our knowledge, this is the first study to demonstrate that the incidence of antepartum DVT had increased in Japan. Obstetricians might have to be strongly encouraged to administer thromboprophylaxis to decrease the incidence of VTE because among women in Japan who were thought to have low risks of VTE, the incidence might have actually increased.

This study included data from 295,961 women, representing 32.2% of the 918,400 women who gave birth in Japan in 2018, that were reported by the Ministry of Health, Labour and Welfare (Japan). Of the women in our study, 20 (0.0068%) died, of whom 18 (90.0%) died in perinatal medical centers. The 184 perinatal medical centers (27.6% of the 666 institutions surveyed) represented 45.3% of all 406 Japanese perinatal medical centers in 2018. According to these data, 47.3 women died during the periparturition period in 2018.

The JAOG reported the deaths of 36 women in 2018, in contrast to the 40, 50, 43, and 47 deaths reported in 2014, 2015, 2016, and 2017, respectively [3]. In the 2018 study, as mentioned, of the 4 women who died of PE, 3 (75.0%) died in perinatal medical centers. These data indicate that 12.4 women died of PE in 2018 study. The maternal deaths from PE in Japan might be more numerous than previously reported [3, 6, 7].

Compared with the antepartum risks of VTE onset during the first, second, and third trimesters, the risk of VTE onset during the first 6 weeks postpartum was significantly higher [16, 17], and the peak of the VTE onset occurred in the first 3 weeks postpartum [17]. In the previous report, the frequencies of antepartum and postpartum VTE onsets were similar (48.9% and 51.1%, respectively) among all women with VTE [16]. In the 2018 study, among the 243 women with VTE, the frequency of antepartum PE onset was lower than that of postpartum VTE onset (35.7% vs. 64.3%), while the frequency of antepartum DVT onset was higher than that of postpartum DVT onset (79.6% vs. 20.4%). The proportion of women with DVT onset was higher than that of women with VTE onset. Thus, the incidence of antepartum VTE onset was higher than that of postpartum VTE onset (67.9% vs. 32.1%; Table 5).

We hypothesized that the frequency of VTE (DVT and PE) onset and death induced by VTE in pregnant women would decrease in 2018 as compared with those reported in the 1991–2000 studies in Japan [6, 7].

The flowchart of the 1991–2000 study is shown in Fig. 1. In the 1991–2000 study in Japan, the data were obtained from 102 institutions [6, 7]. At these institutions, 436,084 women delivered after 22 gestational weeks in 1991–2000. Supplemental Table 1 shows the differences in VTE onset between the data of the 1991–2000 study in Japan [6, 7] and those of the 2018 study. The odds ratio (95% CI) of VTE in the 1991–2000 study are summarized in Table 4.

Of 203 women with VTE in the 1991–2000 study, 127 (62.6%) had DVT and 76 (37.4%) had PE. In the 1991–2000 study, of the 127 women with DVT, 64 (50.4%) had an antepartum onset and 63 (49.6%) had a postpartum onset, and of 76 women with PE, 17 (22.4%) had an antepartum onset and 59 (77.6%) had a postpartum onset. In the 1991–2000 and 2018 studies, the incidence rates of antepartum PE were similar, but the incidence rate of antepartum DVT increased in the 2018 study (Supplemental Table 1). In the 1991–2000 and 2018 studies, the incidences of PE in Japan were similar; however, the incidence of DVT increased from the rate reported in the 1991–2000 study to that reported in the 2018 study (Supplemental Table 1).

In the 1991–2000 study, of the 63 women who had postpartum DVT and 59 women who had postpartum PE, 28 (44.4%) and 9 (15.3%) experienced the onset after vaginal delivery, respectively, and 35 (55.6%) and 50 (84.7%) experienced the onset after cesarean section, respectively. Between the 1991–2000 and 2018 studies, the incidence of postpartum DVT in Japan decreased, and that of postpartum PE decreased. However, these trends were not significantly different (Supplemental Table 1).

The incidence rates of DVT and PE after cesarean section in the 1991–2000 study were similar to those in the 2018 study; however, the incidence rates of DVT and PE after cesarean section decreased. Thus, performing thromboprophylaxis after cesarean section in Japan would be safe and effective (Supplemental Table 1).

In the 1991–2000 study, none of the 127 pregnant women with DVT died; however, of 76 pregnant women with PE, 11 (14.5%) died. The mortality rate from PE was 0.0025% (11/436,084). In 2018 study, the mortality rate of pregnant women with PE was 7.1% (4/56), and the incidence of maternal death from VTE was 0.0014% (4/295,961). The frequencies in the 1991–2000 (*p* = 0.2688) and 2018 studies (*p* = 0.4307) were similar (Supplemental Table 1).

In comparison with the 1991–2000 study, the 2018 study showed lower incidence of VTE after cesarean section but higher incidence of antepartum DVT.

It is possible that the finding of an increase in antepartum DVT might be attributable to the bias of the surveillance questionnaire. In the 1991–2000 study in Japan, the data were obtained from 102 institutions, which included 68 university hospitals and 34 general hospitals [6, 7]. In our study, the 666 institutions included 63 university hospitals (9.5%). However, we suggest that the incidence of antepartum DVT might have increased for four reasons. First, most obstetricians began to pay more attention to antepartum VTE onset and became more skillful at diagnosing DVT after the 2008 publication of guidelines with the recommendation of thromboprophylaxis in Japan. Thus, the number of diagnoses of antepartum DVT would have increased. Second, the number of institutions that routinely performed thromboprophylaxis (especially at antepartum) was small. In comparison with the Western countries, Japan showed a lower proportion of pregnant women with induced VTE because of the lower number of pregnant women with overweight or obesity. Thus, many obstetricians have not used thromboprophylaxis to prevent VTE onset. To decrease incidence of antepartum VTE, an extensive and systematic campaign to encourage antepartum thromboprophylaxis according to the recommendations in Japan [11, 12] may be necessary. Third, the numbers of pregnant women at high risk for antepartum DVT (e.g., women older than 35 and those who became pregnant after assisted reproductive technology) in Japan have increased. Hyperemesis, antepartum bed rest, and ovarian hyperstimulation syndrome are risk factors for VTE. Fourth, antepartum laboratory tests of coagulation and fibrinolysis are not performed to screen for thrombophilia in Japan. Thus, thrombophilia in pregnant women is sometimes not diagnosed before the occurrence of antepartum or postpartum VTE.

In many cases of antenatal VTE, the onset occurred during the third [16, 17] or first trimester [18–20]. Thus, antepartum VTE is a significant concern, and thromboprophylaxis has been recommended [20, 21]. The Royal College of Obstetricians and Gynecologists recommends that women who undergo invasive surgery during pregnancy should be given prophylactic anticoagulation therapy [13]. The 1991–2000 study in Japan had no data about the incidence of perioperative VTE (DVT or PE) during pregnancy [6, 7]. In our study, among the 165 women with antepartum VTE, the incidence rate of perioperative PE during pregnancy was significantly higher than that of perioperative DVT. According to the guidelines about thromboprophylaxis in Japan (Table 1), pregnant women who undergo surgery during pregnancy are at moderate risk for VTE; however, they may have to be considered at high risk to decrease the incidence of perioperative VTE (especially PE) during pregnancy.

Cesarean section is a risk factor for postpartum VTE [22]. The unadjusted relative risk of postpartum VTE after cesarean delivery (versus vaginal delivery) was reported to be 2.6 in 1988 and 1997 in London [4]. In a 2004–2014 study in the United States [10], the incidence of DVT by cesarean section decreased 40% (from 94 to 56 per 100,000 deliveries); however, the incidence of PE caused by cesarean section was not observed to decrease (20, 30, and 23 per 100,000 deliveries in 2012, 2013, and 2014, respectively), neither the incidence of DVT nor that of PE after vaginal deliveries decreased. In Japan, the incidence rates of DVT and PE after vaginal delivery in the 1991–2000 study were similar to those reported in the 2018 study (Supplemental Table 1). In general, pregnant women with a vaginal delivery have lower risks of VTE than those with a cesarean section. In the 2018 study, the incidence of UFH or LMWH administration in women who had PE at/after vaginal delivery was low (Table 5). Thus, thromboprophylaxis after vaginal delivery by women at high risk of VTE should be promoted more extensively among obstetricians to decrease the incidence of VTE after delivery. Furthermore, of the 4 women who died from PE, 2 (one with PE onset during pregnancy without obstetrical operation and the other with PE onset after cesarean section) did not receive UFH or LMWH.

To our knowledge, this is the first study to demonstrate the incidence rates of onsets of other VT and ATE in pregnant women. In the present study, the frequencies were very low (8.8 and 1.4 per 100,000 population, respectively). Furthermore, none of the women died from other VT or ATE. Thus, only few obstetricians might consider the incidence rates of the onsets of other VT and ATE in pregnant women. Further studies are required to determine the risk factors of the onset of other VT or ATE in pregnant women.

Our study had some strength. First, the selection bias of the institutions was low because the surveillance questionnaire was sent to all 2299 hospitals or maternal clinics reported to treat deliveries in Japan. Second, we had access to the data about incidence of perioperative VTE during pregnancy, which was reported by few previous studies. Finally, we investigated the difference in the incidence rate of VTE in Japan between the 1991–2000 and 2018 studies.

This study, however, also had some limitations. First, the surveillance questionnaire did create a potential for some bias. This study might have a recall bias because the institutions that attended to many women with VTE might have more likely responded to the questionnaire (especially on the mortality induced by VTE) than those without a VTE. The information bias by the questionnaire interviewers who determined the study design would be low, as the questions and answer choices of the questionnaires were determined by all authors of this manuscript and checked by the members of the institutional review board. The selection bias from the answers of the obstetricians from the institutions who responded to the questionnaires would be low because the surveillance questionnaire was sent by mail to the chairman, chief, or the substitute of the chairman or chief of all 2299 hospitals or maternal clinics that reported to attend to deliveries in Japan. Meanwhile, obstetricians without experience in the management of pregnant women with VTE might avoid responding to the questionnaires. Thus, the surveillance questionnaire was sent to all 2299 obstetrical institutions in Japan to reduce the selection bias that might induce a low response rate in the present study. Second, we had no detailed information about the patients’ backgrounds, e.g., age, times of previous deliveries, history of thrombophilia or previous VTE, number of gestational weeks at delivery, number of gestational weeks at onset of antepartum VTE, number of postpartum days at VTE onset, and whether thromboprophylaxis was performed. Finally, the data for only 1 year (in 2018) were available. Thus, our results alone could not describe the trend of incidence and mortality of VTE. However, we compared them with the incidence of VTE found in a previous study in Japan.

## Conclusions

Among women in Japan who are thought to have a low risk for VTE during the antepartum period, and especially those with a vaginal delivery by an obstetrician, the incidence of VTE might have increased. More extensive use of thromboprophylaxis for those women with low risks for VTE might decrease the incidence of VTE.

## Supplementary Information

**Additional file 1: Supplemental Table 1**. Difference in the incidence rate of VTE per 100,000 between 1991–2000 and 2018 in Japan

## Data Availability

The datasets used and/or analyzed during the current study will be available from the corresponding author on reasonable request.

## Abbreviations

ATE: Arterial thromboembolism
CI: Confidence interval
DVT: Deep vein thrombosis
JAOG: Japan Association of Obstetricians and Gynecologists
JSOG: Japan Society of Obstetrics and Gynecology
JSOGNH: Japan Society of Obstetrical, Gynecological and Neonatal Hematology
Other VT: Other vein thrombosis
PE: Pulmonary thromboembolism
VTE: Venous thromboembolism
