# Supplementary material for: A retrospective cohort study using a national surveillance questionnaire to investigate the characteristics of maternal venous thromboembolism in Japan in 2018
Source: BMC Pregnancy Childbirth. 2021 Jul 17;21:514. doi: 10.1186/s12884-021-03993-1 (PMC8286571; doi:10.1186/s12884-021-03993-1)
Supplement: Supplementary file 1 — Additional file 1: Supplemental Table 1. Difference in the incidence rate of VTE per 100,000 between 1991–2000 and 2018 in Japan [file 12884_2021_3993_MOESM1_ESM.docx]

**Supplemental Table 1.** **Difference in the incidence rate of VTE per 100,000 between 1991–2000 and 2018 in Japan**

|  | **VTE ^*^** | | | **DVT ^**^** | | | **PE ^**^** | | |
| --- | --- | --- | --- | --- | --- | --- | --- | --- | --- |
|  | 1991–2000 | 2018 | *p-value* | 1991–2000 | 2018 | *p-value* | 1991–2000 | 2018 | *p-value* |
| **Overall** | 36.5 | 71.9 |  | 29.1 | 53.0 | *<0.0001* | 17.4 | 18.9 | *0.6405* |
| **Incidence antepartum** | 18.6 | 49.0 | *<0.0001* | 14.7 | 42.2 | *<0.0001* | 3.9 | 6.8 | *0.0962* |
| **Incidence postpartum** | 28.0 | 23.0 | *0.1294* | 14.4 | 10.8 | *0.1803* | 13.5 | 12.2 | *0.6147* |
| **Incidence after cesarean section** | 97.3 | 63.5 | *0.0214* | 40.1 | 25.1 | *0.1245* | 57.2 | 38.4 | *0.1057* |
| **Incidence after vaginal delivery** | 10.6 | 11.0 | *0.8974* | 8.0 | 6.6 | *0.6404* | 2.6 | 4.4 | *0.2504* |
| **Maternal deaths induced by PE** | 2.5 | 1.4 | *0.4307* | - | - | - | 2.5 | 1.4 | *0.4307* |

VTE, venous thromboembolism; DVT, deep vein thrombosis; PE, pulmonary thromboembolism

*VTE was DVT and/or PE. The simultaneous occurrence of DVT and PE was defined as PE.
